# Supplementary material for: Cancer Patients’ Prehospital Emergency Care: Post Hoc Analysis from the French Prospective Multicenter Study EPICANCER
Source: J Clin Med. 2021 Mar 9;10(5):1145. doi: 10.3390/jcm10051145 (PMC7967166; doi:10.3390/jcm10051145)
Supplement: Supplementary file 1 [file jcm-10-01145-s001.pdf]

Supplementary Table S1. Collaborators and members of the Initiatives de Recherche aux Urgences (IRU) research network who participated in the study.

IRU Local Investigator	Center	City
Patrice SERRE; Noémie MARCHAND	CH FLEYRIAT	BOURG-EN-BRESSE
Marie MAS	CLINIQUE CONVERT	BOURG-EN-BRESSE
Stéphane BLAIN	HOPITAL PRIVE D'AMBERIEU EN BUGEY	AMBERIEU-EN-BUGEY
Hélène KEMORGANT	CH VICHY	VICHY
Aurélie ARNAUD	CHU MONTLUÇON	MONTLUCON
Céline OCCELLI	CHU NICE	NICE
Edouard JACQUET	CHI DES VALLEES D'ARIEGE	FOIX
Hélène QUESTIAUX	CH TROYES	TROYES
Magali KRAIF ; Marc POYEN ; Patrick GERBEAUX	H TIMONE - LA CONCEPTION	MARSEILLE
Hugo LENGLET	BATAILLON DES MARINS POMPIERS DE MARSEILLE	MARSEILLE
Delphine LEVY	CH DU PAYS D'AIX	AIX-EN-PROVENCE
Yves COSSE	CH BAYEUX	BAYEUX
Bastien MALOSSANE	CH AURILLAC	AURILLAC
Stéphane GILLET	H D'ANGOULEME	ANGOULEME
Marc PETER	CHG DE JONZAC	JONZAC
Jean HELIN ; Jeannot RAMAMONJISOA	CH BOURGES	BOURGES
Aurélie AVONDO; Didier HONNART; Benoit SAHUC	CHU DIJON	DIJON
Christophe BARBERIS	CH SAINT-BRIEUC	SAINT-BRIEUC
Abdo KHOURY	CH BESANCON	BESANCON
Eloi NENERT	CH INTERCOMMUNAL DE HAUTE-COMTE	PONTARLIER
Ali FAOUR	CH BERNAY	BERNAY
Jérôme FAURE PONTIER	CH VERNON	VERNON
Béatrice GAIDAMOUR	CH EVREUX	EVREUX
Anastasia DESSENA; Francesca DONATI	CH DREUX	DREUX
Marjorie COUTANT	CH PAYS DE MORLAIX	MORLAIX
Anne LE COAT	HIA BREST	BREST
Alice HURGON	CH COMMINGES PYRENEES CHCP ST GAUDENS	SAINT-GAUDENS
Manon HEBRARD	CHU TOULOUSE	TOULOUSE
Thierry BABET; Rishad VALLLY	CHU PELLEGRIN	BORDEAUX
Pascal BISSOLOKELE	CH STE FOY LA GRANDE	SAINTE-FOY-LA-GRANDE
Julliane BOSC	CH LIBOURNE	LIBOURNE
Simeon SIAGNI	CH DE LA HAUTE GIRONDE	BLAYE
Justine BEREAU ; Sandrine DOJAT	CH SAINT-ANDRE	BORDEAUX
Sophie LEFEBVRE	CHU MONTPELLIER	MONTPELLIER
Myranne LALOUE ; Pauline LEGOFF	CHU RENNES	RENNES
Sophie DABIN ; POUCHARD	CHU TOURS	TOURS
Cédric FALCON; Maxime MAIGNAN	CHU GRENOBLE	GRENOBLE
Ernesto MAIELLO	CH VIENNE	VIENNE

Vivien BRENKMANN; Cyrielle CLAPE; Caroline SANCHEZ	CHU GRENOBLE	GRENOBLE
Pierre ACHACHE	GH DU NORD DAUPHINE	BOURGOIN JALLIEU
Yves DUFFAIT	CH LONS LE SAUNIER	LONS-LE-SAUNIER
Jean FABRE	CH DAX	DAX
Jérôme DIMET	CH MONT DE MARSAN	MONT-DE-MARSAN
Mikael MARTINEZ	CH FOREZ	MONTBRISON
Coralie CHASSIN	CH ROANNE	ROANNE
Hosam HADJADJ, Nesrine BENAOUICHA	CHU NANTES	NANTES
Mathieu OBERLIN	CH CAHORS	CAHORS
Pierre-Arnaud FORT	CH AGEN-NERAC	AGEN-NERAC
Delphine DOUILLET	CHU ANGERS	ANGERS
Oriane VICENZI	CH CHALONS EN CHAMPAGNE - STE MENEHOULD	CHALONS-EN-CHAMPAGNE - STE-MENEHOULD
Pierre-Etienne COLOT	CHU REIMS	REIMS
Anthony MILLET	CH LAVAL	LAVAL
Sabine DAGUERRE	CH PONT A MOUSSON	PONT-A-MOUSSON
Lise BLANCHARD	CH VERDUN HOP ST NICOLAS	VERDUN
Alexandre TANNEAU	CH BRETAGNE SUD	LORIENT
Christophe ROTHMANN	CHR METZ - H MERCY	METZ
Eric GRAVE	CH DUNKERQUE	DUNKERQUE
Céline BORZYMOWSKI	CH VALENCIENNES	VALENCIENNES
Sylvain THIRIEZ	CH ROUBAIX	ROUBAIX
Jean-Baptiste LEZY	CH ARMENTIERES	ARMENTIERES
Romain LECOMTE	CH CAMBRAI	CAMBRAI
Lila ABDELLI	CH TOURCOING	TOURCOING
Quentin RIVIERE	CH BEAUVAIS	BEAUVAIS
Claire HOCHART	CH BETHUNE	BETHUNE
Karine HUMBERT	POLYCLINIQUE DE LA CLARENCE	DIVION
Antoine DE LUCCA	CH CALAIS	CALAIS
Sonia AJIMI	CHU CLERMONT-FERRAND	CLERMONT-FERRAND
Daniel PIC	CH PAUL ARDIER ISSOIRE	ISSOIRE
Guilhem SOLA	CH GUY THOMAS RIOM	RIOM
Adrien PUIG	H DE LANNEMEZAN	LANNEMEZAN
Julie ROSENBLATT	CH DE BIGORRE	TARBES
Pierrick LE BORGNE; Matthieu DELLEMBACH ; Carmen HAMMANN DURR	CHU STRASBOURG HAUTEPIERRE	STRASBOURG
Gautier DROMSON	CH SELESTAT - OBERNAI	SELESTAT
Caroline WITT	CHI DE LA LAUTER	WISSEMBOURG
Kasarra BEN HAMMOUDA	CH COLMAR	COLMAR
Jacques SCHMITT	CH MULHOUSE (GHRMSA)	MULHOUSE
Marine DELAROCHE ; Laurent JACQUIN	CHU LYON (HERRIOT)	LYON
Marion DOUPLAT	CENTRE HOSPITALIER LYON SUD	LYON
Sylvain CHARREYRE	CHU LYON	LYON

Séverine GOSSELIN	CH MACON	MACON
Adrien PICAUD	CH LE MANS	LE MANS
Anaëlle BATICLE	CH CHAMBERY	CHAMBERY
Alban FOREL ; Cécile VALLOT	CH ANNECY	ANNECY
Claire VALLENET	CH ALPES LEMAN	CONTAMINE-SUR-ARVE
Marie-Laurence FIEVET-BROCHOT ; Evelyne DUBREUCQ et Anne-Laure PAQUET	CHU PITIE-SALPETRIERE	PARIS
Pierre-Clément THIEBAUD	CHU ST ANTOINE	PARIS
Jennifer TRUCHOT	CHU LARIBOISIERE	PARIS
Lionel LAMHAUT	CHU NECKER	PARIS
Fred VOISIN; Florence DUMAS	CHU COCHIN	PARIS
Anaïs GUINCESTRE	CHU HEGP	PARIS
Eloïse TRABATTONNI; Jean Luc AIM	GH ST JOSEPH	PARIS
Eric BURGGRAFF	CHU TENON	PARIS
Laurent PEREIRA	CHU BICHAT	PARIS
David BLONDEEL; Céline HOFFMANN	CROIX ST SIMON	PARIS
Madze ANANI MEKLE ; Béatrice DEWEVRE	GH LE HAVRE	LE HAVRE
Quentin FOUBERT; Adrien LEBROZIDEC	CHI CAUX VALLEE DE SEINE	LILLEBONNE
Luc-Marie JOLY ; Mélanie ROUSSEL	CHU ROUEN	ROUEN
Fabrice BOISHARDY; Flore GEERLANDT	CHIC ELBEUF-LOUVIERS	ELBEUF - LOUVIERS
Maud FLAMBARD	CH FONTAINEBLEAU	FONTAINEBLEAU
Omar BEKHODJA; TOUMANI Samir	GH DE L'EST FRANCILIEN	COULOMMIERS
Chloé HOZE	H PRIVE MARNE CHANTEREINE	BROU-SUR-CHANTEREINE
Laurence SZTULMAN	GH DE L'EST FRANCILIEN	MEAUX
Sandra BERNARD	H F. QUESNAY	MANTES-LA-JOLIE
Laurence BERTON	CH VERSAILLES	VERSAILLES
Thomas LEREDU; Olivier RICHARD; Ludovic DALLE	CH VERSAILLES-LE CHESNAY	VERSAILLES
Mathieu VIOLEAU; Pierre-Alexis BOURRY	CH NIORT	NIORT
Catherine VELLY; Helene JOUDRIER	CH TOULON – LA SEYNE	TOULON
Cyril COUILLARD, Jean TIDA	CH SUD VENDEE	FONTENAY-LE-COMTE
Philippe FRADIN; Emelyne CWICKLINSKI	CHD VENDEE LA ROCHE/YON	LA ROCHE-SUR-YON
Claudie AUDRAIN	CH LUCON	LUCON
Pascal GABY	CH G.CLEMENCEAU	MONTAIGU
Nicolas MARJANOVIC; Carine SUROUX	CHU POITIERS	POITIERS
Christine VALLEJO	CHU LIMOGES	LIMOGES
Marie-Paule BITAR	CH DE REMIREMONT	REMIREMONT
Ayoub TOUIHAR	CH AUXERRE	AUXERRE
Trung Hung TA ; Andrianjafy HERY	CHG LONGJUMEAU	LONGJUMEAU
Laurène VASSEUR	CORBEIL-ESSONNES	CORBEIL-ESSONNES
Sébastien BEAUNE	CHU AMBROISE PARE	BOULOGNE
Gaêlle LE BAIL	CHU RAYMOND POINCARE	GARCHES
Siva PRABAKAR	CHU BEAUJON	CLICHY

Julie CELERIER	H FRANCO-BRITANIQUE	LEVALLOIS-PERRET
Nicolas JAVAUD	CHU LOUIS MOURIER	COLOMBES
Anna BOUCHARA	CHI R.BALLANGER	AULNAY-SOUS-BOIS
Romain DUFAU; Luis SEGURA	CHU JEAN VERDIER	BONDY
Emmanuelle ZAMPARINI	CH MONTFERMEIL	MONTFERMEIL
Sheila GASMI, Frédéric ADNET	CHU AVICENNE	BOBIGNY
Astrid GUILLOIS	HOPITAL DE DELAFONTAINE	SAINT-DENIS
Stéphane DIEZ	CHU BICETRE	LE KREMLIN-BICETRE
Mohamed KHALID; Lionel NAKAD	CHU HENRI MONDOR	CRETEIL
Layla YAHYAOUI	CHI CRETEIL	CRETEIL
Julie ZUNDEL; Corine BERGERON	CHI VILLENEUVE ST GEORGES	VILLENEUVE-SAINT-GEORGES
Marie-Valérie BOURHIS	HIA BEGIN	SAINT-MANDE
Xavier BAERMAN; Catherine LEGALL; Nasro BENREZZAK	CH ARGENTEUIL	ARGENTEUIL
François DUPAS; Sandrine GOULVENT; Véronique SANH	CH RENE DUBOS	PONTOISE
Laurie REAUX; Marie-Laure DEVAUD; Yves DUMORA	CH BEAUMONT SUR OISE	BEAUMONT-SUR-OISE
Maxime CANTREAU; Nathalie ROUDIAK; Mustapha YOUSSEF	CH GONESSE	GONESSE
Sylvie POTHAULT; Céline MAISONDIEU	CHU FORT DE FRANCE	MARTINIQUE
Guillaume MICHOT; Flore PINEAU	CHU SUD REUNION BELLEPIERRE	REUNION
Maylis DOUINE; Mamadou SOW	CH CAYENNE	CAYENNE
Yann-Erick CLAESSENS	CH PRINCESSE GRACE	MONACO
Kouchiar AZARNOUSH	CH FRIBOURG	FRIBOURG
